# Comparative genomics of *Lactobacillus kefiranofaciens* ZW3 and related members of *Lactobacillus*. spp reveal adaptations to dairy and gut environments

**DOI:** 10.1038/s41598-017-12916-0

**Published:** 2017-10-09

**Authors:** Zhuqing Xing, Weitao Geng, Chao Li, Ye Sun, Yanping Wang

**Affiliations:** 1 Key Laboratory of Food Nutrition and Safety, Ministry of Education, Food Engineering and Biotechnology Institute, Tianjin University of Science & Technology, Tianjin, 300457 China; 2 Chinese medical college of TJUTCM, Tianjin University of Traditional Chinese Medicine, Tianjin, 300193 China

## Abstract

It is important for probiotics that are currently utilized in the dairy industry to have clear genetic backgrounds. In this study, the genetic characteristics of *Lactobacillus kefiranofaciens* ZW3 were studied by undertaking a comparative genomics study, and key genes for adaptation to different environments were investigated and validated *in vitro*. Evidence for horizontal gene transfer resulting in strong self-defense mechanisms was detected in the ZW3 genome. We identified a series of genes relevant for dairy environments and the intestinal tract, particularly for extracellular polysaccharide (EPS) production. Reverse transcription-qPCR (RT-qPCR) revealed significant increases in the relative expression of *pgm*, *ugp*, and *uge* during the mid-logarithmic phase, whereas the expression of *pgi* was higher at the beginning of the stationary phase. The enzymes encoded by these four genes concertedly regulated carbon flux, which in turn modulated the production of EPS precursors. Moreover, ZW3 tolerated pH 3.5 and 3% bile salt and retained cell surface hydrophobicity and auto-aggregation. In conclusion, we explored the potential of ZW3 for utilization in both the dairy industry and in probiotic applications. Additionally, we elucidated the regulation of the relevant genes involved in EPS production.

## Introduction

Lactic acid bacteria (LAB) are gram-positive bacteria that naturally occur in the environment and are closely associated with humans, animals and plants. Due to recent advances in LAB research, LAB genotype information in combination with phenotypic characteristics have been applied in the food and medical industries to explore potential biological functions for development and various applications. Genome-wide sequencing has been performed for more than 200 strains of LAB, and many of these strains, such as *Lb*. *acidophilus* NCFM^1^, *Lb*. *plantarum* WCFS1^2^, and *Lb*. *rhamnosus* GG^3^, are commonly used in industrial applications and have confirmed probiotic functions. Numerous comparative genomics investigations of *Lactobacillus* spp. have been conducted. Some of these only targeted a single species in revealing the core and variable genome such as *Lb*. *rhamnosus* ^4^, *Lb*. *sakei* ^5^, and *Lb*. *paracasei* ^6^. Other comparative genomic studies involving various species of *Lactobacillus* spp. have been conducted to expand their biotechnology potential^7,8^. LAB genomes are relatively small and have low GC content. Furthermore, common metabolic pathways among strains have been identified; thus, different strains share relatively close evolutionary relationships^9^. However, due to differences in living conditions, LAB strains have evolved, thereby increasing their genetic diversity^10^. The correlation between gene loss and niche adaptation was examined by growing nine *Lb*. *casei* strains isolated from various sites^11^. The identification of key genes in the LAB genome that were related to adaptation to the intestinal tract or a milk environment may facilitate the determination of whether a strain has the potential for probiotic or dairy applications in industry.

*Lb*. *kefiranofaciens* contributes to kefiran formation and plays a role in a wide range of probiotic functions, such as immunomodulation^12^, anti-allergic potential^13^, and modulation of the gut microbiota^14^. However, studies on this species at the genomic level are limited; for example, one investigation involved screening the biotechnology potential of 213 strains, but less information was provided for this species^7^.

ZW3 is the first *Lb*. *kefiranofaciens* strain that has been completely sequenced. ZW3 was originally isolated from the traditional functional fermentation product kefir and produces high levels of extracellular polysaccharide (EPS)^15,16^. To obtain current and accurate genetic information about ZW3, its genome was re-annotated, and its genetic characteristics were analyzed in greater detail in this study. Moreover, to explore its relationship with other LAB strains at the genome level, the ZW3 genome was compared with those of five other *Lactobacillus* strains. Moreover, its potential use as a probiotic or dairy starter strain was examined. Finally, relevant genes for its survival in dairy and intestinal environments were analyzed and verified.

## Results and Discussion

### General features of the *Lb*. *kefiranofaciens* ZW3 genome

**Figure 1:**
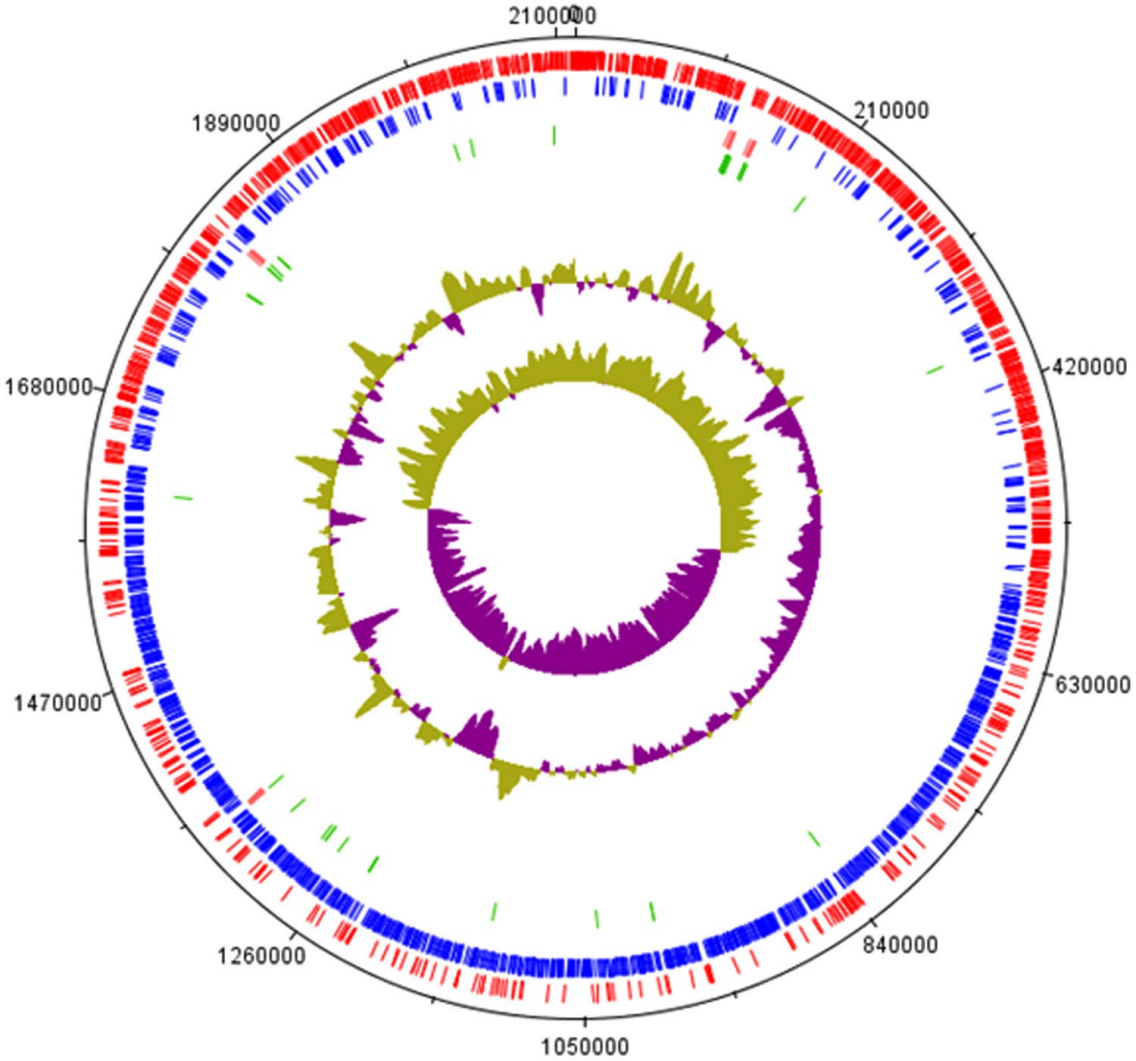
Circular map of the *Lactobacillus kefiranofaciens* ZW3 genome. Genomic features, moving from the periphery to the center of the map: 1. forward CDSs (red); 2. reverse CDSs (blue); 3. rRNA genes (orange); 4. tRNA genes (green); 6. GC% plot; and 7. GC skew.

The ZW3 genome was re-annotated using the RAST and Basys platforms and verified using the Gene PRIMP system. After manual curation, we obtained credible genome-wide sequence annotation information (Fig. 1). The genomic length of the *Lb*. *kefiranofaciens* ZW3 genome was 2,113,023 bp, and the GC content was 37.7%, with 2,181 DNA coding sequences (CDS). In a 2011 study, we only identified 1,908 CDSs in the ZW3 genome^15^. However, since then, new whole-genome sequences have been uploaded to the database, and thus more information was available for exploration in this study; hence, we carried out the re-annotation. Next, we classified the function of CDSs in the ZW3 genome (Fig. S1). In terms of bacterial cell materials and energy supply, the number of genes involved in protein metabolism and carbohydrate metabolism was the highest (186 and 165, respectively). Moreover, numerous genes (71) were related to cell wall and cell membrane synthesis, of which 16 genes were involved in EPS synthesis and 12 in sialic acid metabolism. Sialic acid is a type of natural sugar-acid compound that commonly occurs in the human intestinal tract^16^, milk^17^, and certain viscous environments. Jamila *et al*.^18^ first discovered the sialic acid metabolic gene cluster in *Lb*. *sake*. The gene cluster is assumed to aid in *Lb*. adaptation to viscous environments. Therefore, we inferred that genes associated with sialic acid metabolism in ZW3 are important for its survival in kefir, which is a notably viscous environment. The comparison of old and new information about the genes involved in sialic acid and extracellular polysaccharide synthesis is shown in Table S1. We found that the previous annotation data was unclear, such as annotations of the genes *WANG_1286*, *WANG_1287*, *WANG_1290*, *WANG_1291*, and *WANG_1295*-*WANG_1299*. After re-annotation, the accuracy of the annotation data was significantly improved, which may be helpful to future targeted studies. Some genes were not detected in the previous annotation data; for example, two phosphotransferase systems related to sialic acid synthesis were not annotated. Interestingly, the gene encoding α-amylase was detected during re-annotation; it showed 99% sequence similarity to their orthologs in two other *Lb*. *helveticus* strains, allowing us to infer that ZW3 may have the ability to utilize starch. Previously, this gene was annotated with the marker “Neopullulanase”, which easily led to its omission from functional screening.

### Whole-genome phylogeny, comparative genomics, average amino acid identity (AAI) analysis, and whole CDS Venn diagrams

A phylogenetic tree containing ZW3 and 74 other *Lactobacillus* strains, including a *Streptococcus infantarius* strain, was constructed based on core gene sequences using EDGAR (Fig. S2). In terms of genetic distance, ZW3 was most closely related to the strain *Lb*. *kefiranofaciens* DSM5016, followed by *Lb*. *helveticus*, *Lb*. *acidophilus*, and *Lb*. *amylovorus*. From species of *Lb*. *helveticus*, we selected three representative strains (R0052, H9, and H10) whose sequence showed good integrity. In addition, we also selected a commercial probiotic strain of *Lb*. *acidophilus* La14 (Table 1) for comparative analysis. As expected, the genomes of the H9 and La14 strains were relatively small, and the other four strains were approximately 2–2.25 Mb in size. According to the genomic data *Lactobacillus* genus, estimated genome sizes ranged from 1.23 Mb (*Lb*. *sanfranciscensis*) to 4.91 Mb (*Lb*. *parakefiri*)^19^, and the number of CDSs ranged from approximately 1,700 to 2,800^20^. Genome size and the number of CDSs in these six strains were all moderate. Moreover, the number of CDSs in ZW3 and DSM5016 were higher than the other strains, indicating more functions.

**Table 1:** Genome information on various *Lactobacillus* strains.

Strain	Genome size (Mb)	GC (%)	CDS number	RNA number
ZW3	2.11	37.7	2,181	72
H9	1.87	37	1,887	72
H10	2.14	36.79	2,049	74
R0052	2.13	36.8	1,980	73
DSM5016	2.25	37.2	2,215	53
La14	1.99	34.7	1,874	73

In homologous species, amino acid sequences are more strongly conserved than nucleotide sequences^21^. Here, the AAI was calculated for each strain, and the results are shown as a matrix (Fig. S3). The AAIs for ZW3 and DSM 5016 were the highest (99.72%), which was indicative of their close evolutionary relationship. The AAI of ZW3 and three strains of *Lb*. *helveticus* was approximately 87%, indicating large amino acid differences in the core genes among different species. We also conducted ANI analysis (Fig. S4), which validated the AAI findings. We identified the pangenome (a 3,904-gene family) and the core genome (907 genes) of the six strains. Orthologous genes of the core genome were primarily related to DNA and RNA metabolism, carbohydrate metabolism, and cell division processes. These genes are generally essential for the growth and reproduction of organisms and constitute the basic life framework of the six strains, which are relatively conserved in *Lactobacillus* spp.

**Figure 2:**
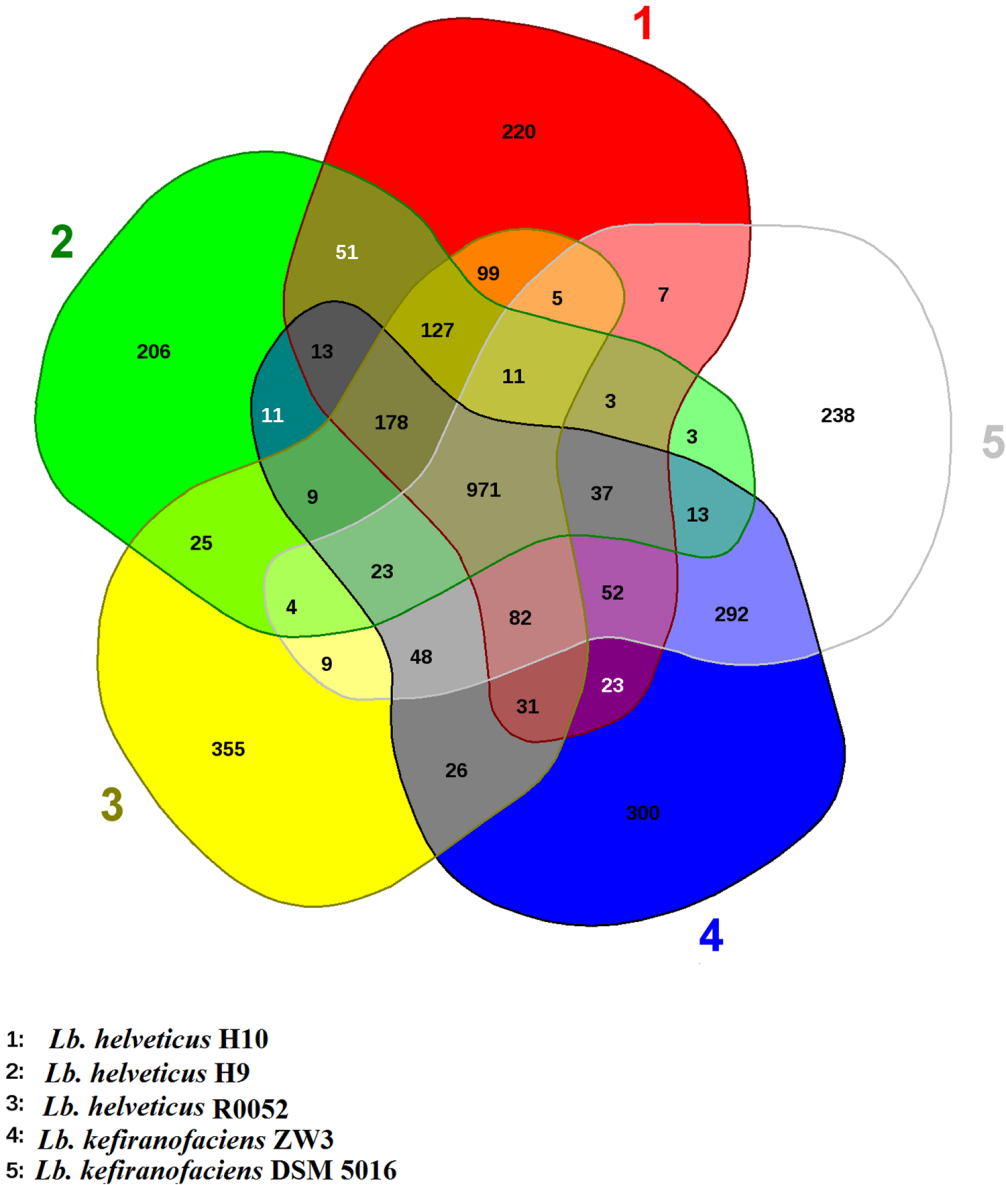
Venn diagram of CDS numbers for *Lactobacillus* spp.

**Figure 3:**
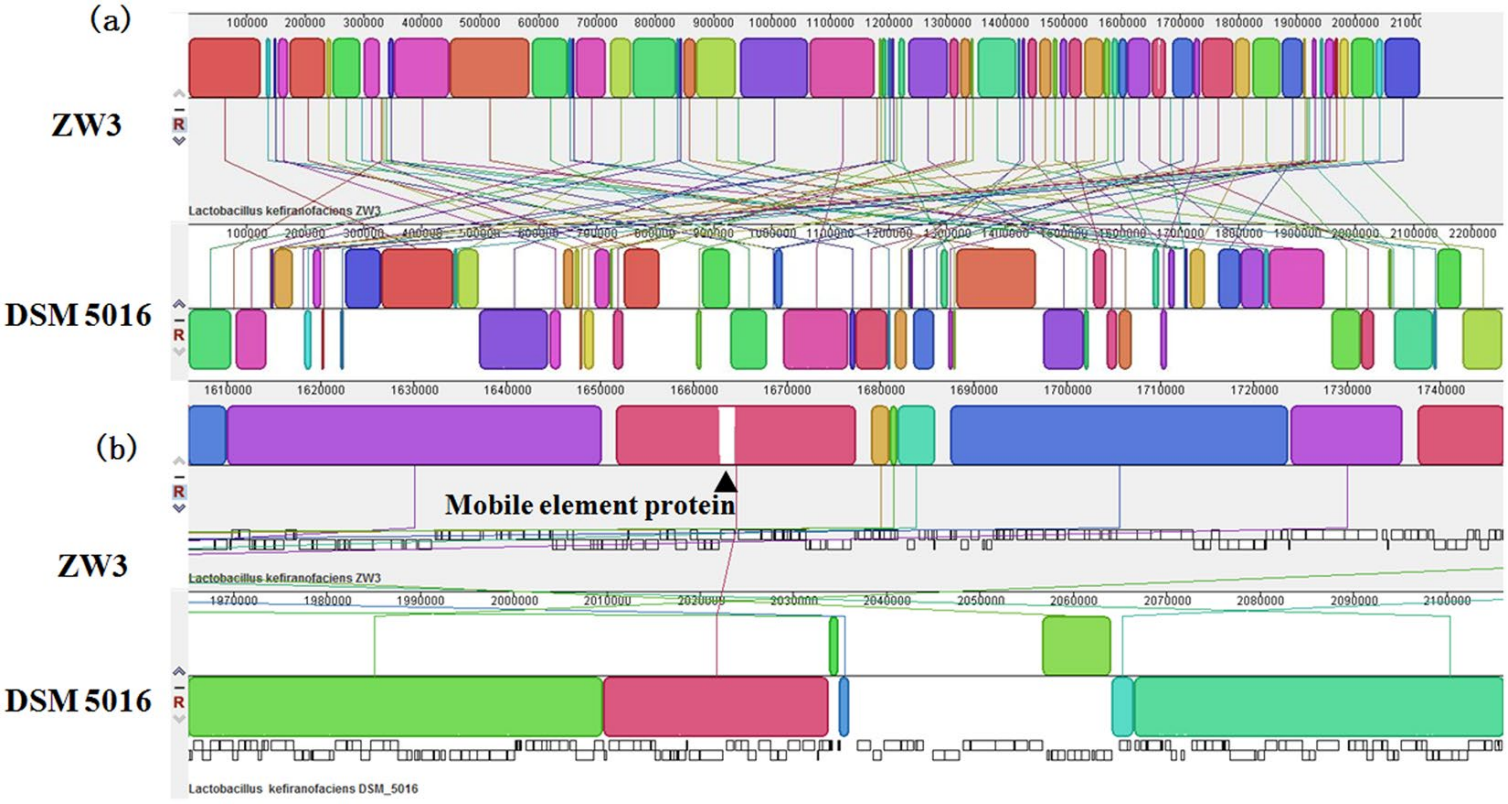
Alignment of the *Lactobacillus kefiranofaciens* ZW3 genome with the *Lactobacillus kefiranofaciens* DSM 5016 genome (**a**. whole-sequence alignment; **b**. partial alignment results).

Because the genetic distance for La 14 was relatively large, we analyzed the gene distribution of five strains, namely, ZW3, DSM 5016, R0052, H9, and H10, by constructing Venn diagrams (Fig. 2). The highest number of unique genes was observed in R0052 (355), followed by ZW3 (300), indicating that these two strains are more diverse at the genetic level. The unique genes in ZW3 comprised 28 hypothetical protein genes, two transposase genes, mobile element protein genes, polysaccharide synthesis proteins, bile salt hydrolases, restriction endonucleases, and specific anchoring proteins. These genes contribute to specific physiological functions in ZW3. Full chromosome alignments of ZW3 and DSM 5016 were also performed (Fig. 3). Analysis revealed a mosaic pattern of homology that was organized in local collinear blocks (LCBs) in both ZW3 and DSM 5016 (Fig. 3a). Evidently, a significant portion of the genetic information in these strains has been conserved, as the majority of the LCBs were shared by both. Moreover, chromosomal rearrangements apparently occurred rather frequently, as the number of LCBs demonstrating changes in their relative genomic position was high but their lengths were short. Additionally, we detected several inversions in the DSM 5016 genome when ZW3 was used as reference. However, numerous differences between ZW3 and DSM 5016 were also detected (Fig. 3b), which were mainly related to mobile protein sequences. According to the combined annotation results, there were 102 mobile element proteins and 7 transposases in ZW3, but only 34 mobile element proteins and 5 transposases were identified in DSM 5016. A higher number of gene fragments were apparently lost during ZW3 genome evolution. Additionally, higher GC content is a sign of rapid ongoing evolution in this particular species^9^. The GC content of the ZW3 genome was slightly higher than that of the DSM 5016 genome, which indirectly verified the results previously described.

### Genomic islands (GIs), multiple restriction-modification (RM) systems, and clustered regularly interspaced short palindromic repeats (CRISPR) regions

GIs are sites of horizontal gene transfer (HGT) that reveal important features of plasticity of a bacterial genome and are primarily linked to gene gain processes^22^. The results obtained with islandViewer are shown in Figure S5. Fourteen GIs were identified in ZW3, and among the six strains, the highest number of GIs was observed in R0052 (29). GI length in ZW3 ranged between 4,344 bp and 31,685 bp, and the total length of all GIs was 151,318 bp, representing 7.16% of the whole genome. However, this value was less than that of DSM 5016 (8.3%), H10 (8.45%), and R0052 (15.79%). Furthermore, GI 7 in ZW3 showed 99% identity to the GIs in DSM 5016 as well as 97% consistency with GI 5 of H9. Moreover, the sequences of H9 and H10 showed 98% similarity to GI 8 in ZW3. GI 12 in ZW3 was 89% identical to GI 1 in the La 14 genome. These findings indicate a high degree of sequence conservation among GIs in *Lactobacillus* spp.

The RM system is a self-defense mechanism that is prevalent in bacteria that prevents and controls phage infections. Variations in the number and type of RM systems between commensal and dairy LABs have been reported^23^. Genes of the type III RM system may constitute a signature for milk adaptation^9^. A type III restriction endonuclease system in ZW3 (also in DSM5016), as well as type I (3), type II (3), and type IV (1) RM systems were identified. The number of RM systems was higher than that observed in R0052, H9, H10, and La 14, whereas DSM 5016 showed an additional type I RM system than that detected in ZW3. Phage infections are highly probable during traditional kefir milk fermentation due to the use of unsterilized raw milk and the open environment during the inoculation process. This situation may promote the selection of phage-resistant strains, which would explain why the RM system in ZW3 was more diversified. Another mechanism of phage resistance, CRISPR, was also detected in the six strains. According to a previous study^24^, the CRISPR-Cas and RM systems are compatible, cleave invading DNA, and provide bacteria with increased phage resistance. The CRISPR-Cas system is capable of cleaving phage DNA that was previously methylated by the RM system. ZW3 harbored two unique CRISPR loci, each with 4 and 3 spacers, which is fewer than in the other strains. Hence, the RM system may play more important roles than CRISPR-Cas in strain ZW3.

### Growth-related genes in milk or dairy products

Some LABs secrete bacteriocin or antimicrobial peptides such as nisin, which has been widely used as a food preservative, to resist adverse environmental impacts. Helveticin J bacteriocin in ZW3 showed 100% sequence similarity with DSM 5016. However, helveticin J was also found in three other strains of *Lb*. *helveticus*. Helveticin J reportedly belongs to the class III bacteriocins, which have a narrow antibacterial spectrum and low thermal stability^25^. To investigate whether the Helveticin J in ZW3 is inhibitory or sterilizing to common pathogens, we employed a number of common pathogens as indicator bacteria, including *E*. *coli*, *E*. *aerogenes*, *M*. *luteus*, and *B*. *subtilis*. After adjusting the pH to neutral and removing hydrogen peroxide, the ZW3 fermentation supernatants did not demonstrate inhibitory functions against the indicator bacteria, corroborating the results of a previous study^25^. To adapt to growth in milk, LAB effectively uses lactose or galactose as carbon source and hydrolyzes casein and whey protein to promote growth and metabolism and to provide the necessary raw materials and energy substrates. All six strains contained enzymes that were related to lactose and galactose supersession with multiple copies to permit full nutrient use in a dairy environment (Table S4). Interestingly, we only found oligofructose and raffinose metabolic systems in La 14, DSM 5016, and ZW3. Fructooligosaccharides are probiotics that stimulate the growth and activity of *Bifidobacterium* spp. and *Lactobacillus* spp. in the intestine^26^. Therefore, we inferred similarity between DSM 5016 and ZW3 as well as La 14, as these strains are capable of adapting to dairy and animal or human intestinal environments. In another study, we demonstrated colonization by ZW3 in the mouse gut (data unpublished). In terms of protein hydrolysis systems, all six strains had similar proteases and aminopeptidases, but the ZW3 and DSM strains lacked a deblocking aminopeptidase, which was present in the other strains. Cheese made with *Lb*. *helveticus*, in which deblocking aminopeptides were knocked out, tastes bitter and is harder than cheese made with wild-type bacteria^27^. Similarly, we found that mozzarella made using ZW3 was much harder than that made with commercial strains (data unpublished).

### Genes involved in survival in the gastrointestinal tract (GIT)

The GITs of animals and humans contain bile, and the bile concentration in the human GIT is between 0.1% and 0.5%, depending on individual differences^28,29^. Most probiotics survive in the bile environment because these express bile salt hydrolase (BSH), which hydrolyzes amide bonds between amino acid groups and cholesterol^30^. We detected two *bsh* genes (*peg*.*857* and *peg*.*858*) and two bile salt transporters (*peg*.*859* and *peg*.*860*) in the ZW3 genome. Using BLASTP, we determined the similarity of *peg*.*857* to the *bsh* gene in *Lb*. *johnsonii* to be 96%. Moreover, the similarity of *peg*.*858* to another *bsh* gene in *Lb*. *johnsonii* was 86% and was 85% for *Lb*. *gasseri* ATCC 332333, which demonstrates excellent tolerance to 0.25% bile^31^. Both *peg*.*859* and *peg*.*860* belonged to the co-transporter superfamily but were quite different from the other species of the *Lactobacillus* genus. Under acid stress, H+ -ATPase^32^, the urease system^33^, and amino acid decarboxylation^34^ act as the main regulatory mechanisms that maintain the internal pH of cells within physiological limits. A pH homeostasis is maintained by discharging H+ from cells, which is dependent on H+ -ATPase^35^. To inspect the acid tolerance of ZW3, the H+ -ATPase (*peg*.*510*) gene was detected, which showed 90% homology to the gene in *Lb*. *crispatus*. Decarboxylation systems consist of a decarboxylase and a precursor/product transmembrane exchanger^36^. Decarboxylase enzymes function in cooperation with an amino acid/amine antiporter system, which moves amines out of the cell in exchange for extracellular amino acids. The massive extrusion of the basic amine product coupled by the uptake of the amino acid substrate may also contribute to an increase in the pH of the extracellular medium^37,38^. We also identified two other genes involved in amino acid decarboxylation in ZW3, namely, ornithine decarboxylase (*peg*.*773*) and amino acid permease (*peg*.*774*), which were also highly homologous (82% and 95%, respectively) to the gene in *Lb*. *acidophilus*, which is involved in acid tolerance^39^. Therefore, we inferred a similar function in terms of acid tolerance in ZW3. To further understand acid and salt tolerance in ZW3, we conducted *in vitro* experiments. ZW3 demonstrated a certain degree of tolerance at pH 3.5, but did not survive in 0.3% bile salt; however, this strain was resistant to a high bile salt concentration (3%). According to previous reports, *Lb*. *gasseri* has lower bile salt tolerance under acidic conditions than under neutral conditions^31^. Thus, we suspect that high bile salt concentrations neutralized the pH of the medium, thereby allowing ZW3 to exhibit bile salt tolerance.

**Figure 4:**
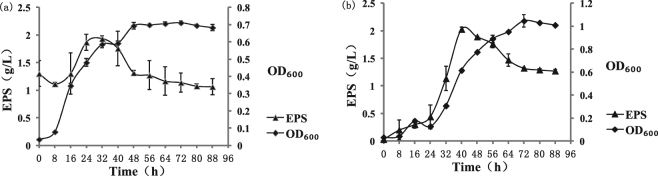
Growth and EPS production curves for *Lb*. *kefiranofaciens* ZW3 (**a**: Whey medium; **b**: modified MRS medium).

Surface protein adhesion plays a key role in the successful colonization of probiotics in the GIT. ZW3 produces high amounts of EPS^40^, which influences bacterial adhesion and survival^41,42^. In our preliminary study, the yield and composition of EPS significantly differed under various medium conditions (data unpublished). Therefore, we investigated the growth curve and EPS-producing curve of ZW3 under two different culture conditions (in whey medium and modified MRS medium; Fig. 4). The logarithmic growth phase of ZW3 was relatively short in whey medium (from 8 h to 48 h; Fig. 4a), and the final OD_600_ = 0.7. However, a longer logarithmic period was observed in the modified MRS culture (from 8 h to 72 h), and the final OD_600_ = 1.0 (Fig. 4b). Whey medium contains 1.3 g/L of polysaccharides at 0 h (starch derived from milk powder). Because ZW3 itself has α- amylase, it can hydrolyze starch; therefore, from 0 h to 8 h, ZW3 mainly consumed starch. In terms of EPS yield, the amount of EPS significantly increased from 16 h to 32 h in whey medium and then decreased, indicating that EPS accumulation was at its peak (1.29 g/L–1.93 g/L) at 32 h when ZW3 was cultured in whey medium. In modified MRS medium, EPS produced by ZW3 accumulated as biomass increased until 40 h, reaching a peak value of 2.03 g/L and then decreasing. These growth and EPS-producing curves indicate that ZW3 exhibits low EPS production during the early logarithmic phase (ELP), whereas highest levels of accumulation occur during the mid-logarithmic phase (MLP) in both media. However, the amount of EPS was significantly reduced at the beginning of the stationary phase (BSP).

**Figure 5:**
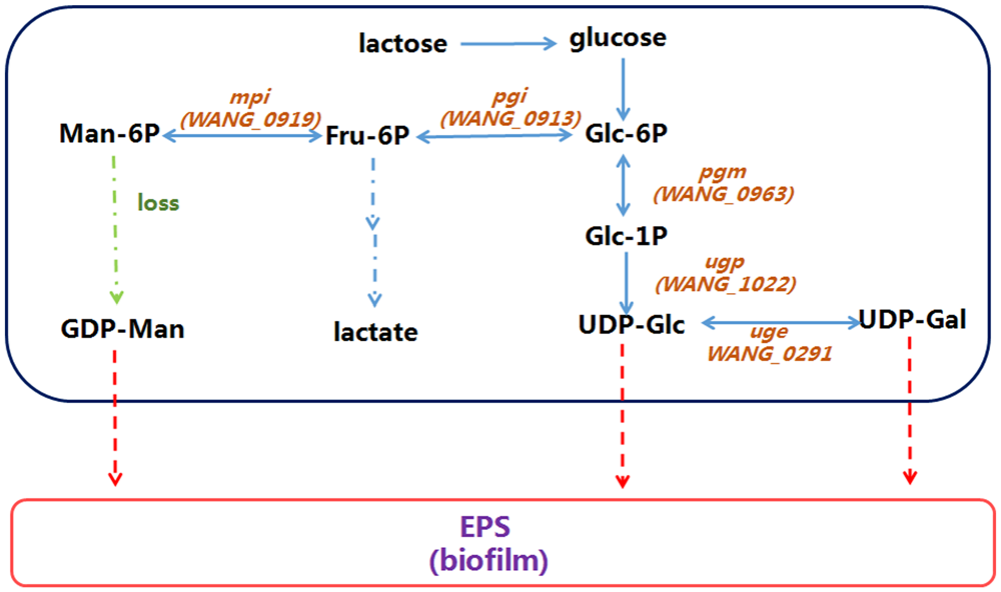
The proposed biosynthesis pathway for EPS production according to KEGG analysis.

We previously confirmed that the medium exerts a significant effect on ZW3 growth status and EPS production, although ZW3 demonstrated the same trend for EPS production at different growth phases. Different components of the medium affect the activity of related enzymes in the EPS synthesis pathway such as that in *Lb*. *bulgaricus* NCFB2772^43^. Thus, the differences in enzyme expression levels in EPS synthesis-related metabolic pathways in various media are confusing. Moreover, it is not clear whether the expression of specific enzymes is up- or downregulated. Therefore, we conducted an analysis of these relevant enzymes. We investigated metabolic pathways in ZW3 using the KEGG database (Fig. 5) by exploring the Leloir pathway^44,45^, which is related to EPS production in LAB. The number of monosaccharide nucleotides impacts the composition and yield of EPS^46,47^. The monosaccharide composition of EPS in ZW3 consists of mannose, galactose, and glucose. Five enzymes were associated with UDP-glucose, UDP-galactose, and GDP-mannose in the ZW3 genome, including glucose-6-phosphate isomerase (*pgi*), α-phosphoglucose mutase (*pgm*), UDP-glucose pyrophosphorylase (*ugp*), UDP-galactose 4-epimerase (*uge*), and mannose-6-phosphate isomerase (*mpi*). However, no enzymes that convert 6-phosphate mannose to GDP-mannose in the GDP-mannose synthesis pathway were detected in ZW3. A previous report similarly indicated the lack of a corresponding enzyme system in *Lb*. *paracasei* LC2W, suggesting that the mannose component of its EPS may be derived from the medium, mannose-6-phosphate, or epoxidose^48^. Thus, we only investigated the catalytic fructose-6-phosphate to mannose-6-phosphate step mediated by *mpi*.

**Figure 6:**
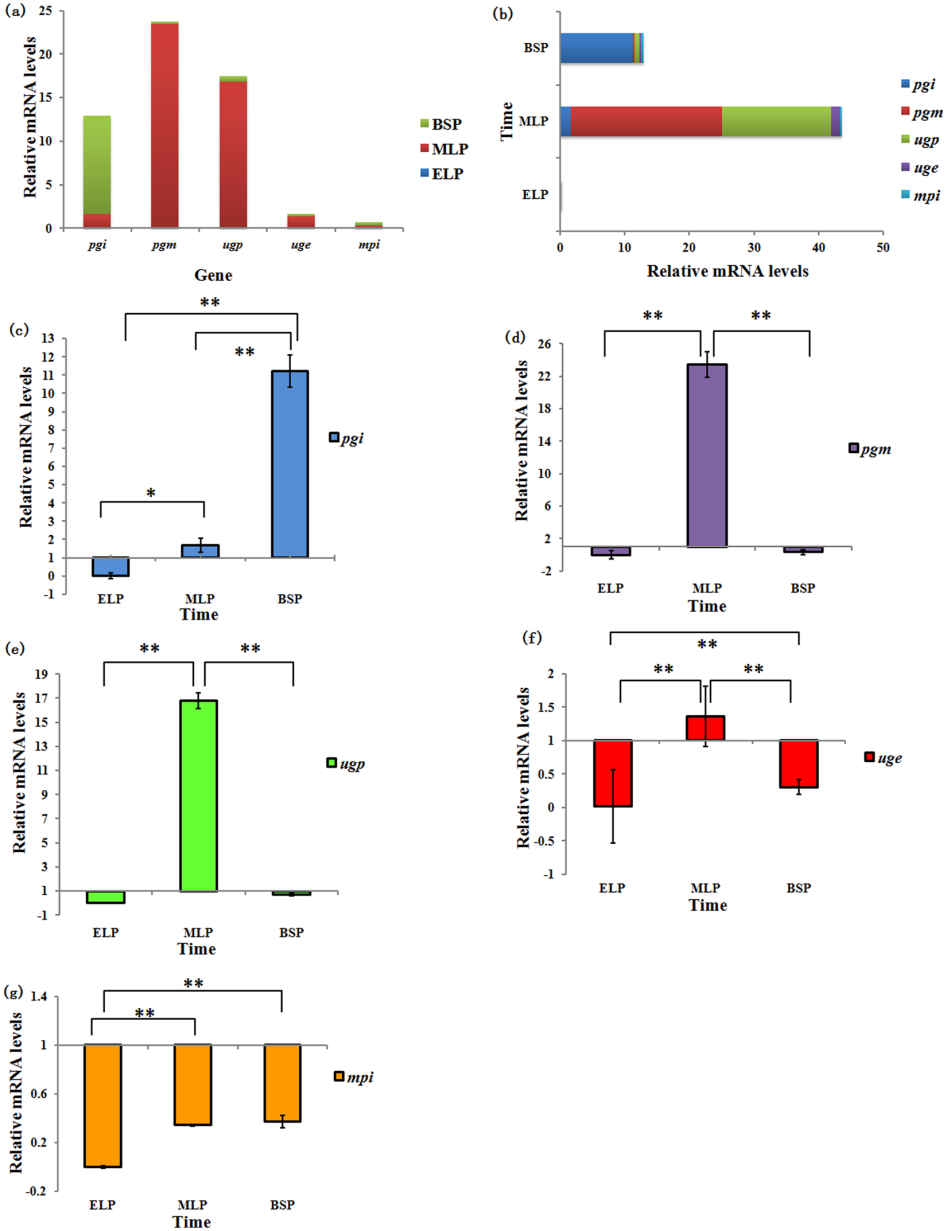
The relative expression levels of each gene at different time points (**a**: the relative expression of one gene at different time points; **b**: the relative expression of different genes at one time point; **c**: the relative expression of *pgi*; **d**: the relative expression of *pgm*; **e**: the relative expression of *ugp*; **f**: the relative expression of *uge*; **g**: the relative expression of *mpi*). Values are expressed as the mean ± standard deviation (S.D.) (n = 3). *P < 0.05, **P < 0.01 compared to another time point.

Next, we detected differences in the expression of the five enzymes mentioned above in two different media at three growth phases. A whey medium cultured group was used as control (Fig. 6). For the same growth phase, relative gene expression significantly differed (Fig. 6a). The highest relative expression during the three growth phases was observed for *pgm*, and the lowest expression was observed for *mpi*, indicating that *pgm* is more positively regulated than *mpi* in modified MRS. *pgm* expression levels during the logarithmic phase were 98.6% of the total expression. Compared to the relative *pgm* expression levels in ELP and BSP, *pgm* demonstrated significant positive regulation in MLP (Fig. 6d). *Pgm* has a significant impact on EPS synthesis, and increasing *pgm* activity significantly increases EPS production^49,50^. *Pgm* catalyzes the conversion of glucose-6-phosphate to glucose-1-phosphate, thereby directing carbon flux to the UDP-glucose synthetic pathway. Therefore, we hypothesized the role of *pgm* to be that of a “switch” during MLP in modified MRS medium, thereby contributing to EPS accumulation. Interestingly, *pgi* differs from *pgm*, *ugp*, and *uge* by showing its highest relative expression during BSP (up to 87% of the total relative expression), as shown in Fig. 6c. Analysis of the active site and conserved domains indicated that *pgi* consists of SIS1 and SIS2 and is relatively conserved. Glucose-6-phosphate was converted to fructose-6-phosphate by *pgi* and then entered glycolysis. Thus, carbon flux was dominated by *pgi*, which primarily flows to the glycolytic pathway, during BSP. This has been verified in *Lactobacillus casei* ^51^ in NADH oxidase overexpression strains, which exhibited reduced lactate and improved EPS production. Thus, our results suggest that the decline in EPS production during BSP was related to the flow of carbon to the glycolytic pathway.

Total relative gene expression was highest during MLP (Fig. 6b). Furthermore, the change in the relative expression of *ugp* was very similar to that observed for *pgm*, which significantly improved during MLP compared to the other two phases (Fig. 6e). PHYRE2 analysis indicated that the UGP of ZW3 was composed of 299 amino acids that belonged to a hydrophilic protein that contained an ADP-binding site, an ATP-binding site, and four glucose-1-phosphate sites. *ugp* affects EPS synthesis in both fungi^52^ and bacteria^53^. UGP catalyzes the synthesis of UDP-glucose by glucose-1-phosphate and UTP molecules, which is the next step in the catalytic reaction performed by *pgm*, directly providing precursors for EPS production. *Uge* catalyzes the conversion of UDP-glucose to UDP-galactose, which is the source of galactose for EPS, in this metabolic pathway. UGE of ZW3 was folded into two different domains containing four active sites, multiple NAD^+^ binding sites, substrate binding sites, and homodimer binding sites. After knocking out *uge*, mutant growth was affected^54^, and EPS production was reduced^55^, with no EPS-induced changes in morphology^56^. Here, we tested *uge* expression levels during MLP, which were significantly higher than in BSP (Fig. 6f), indicating the important role of *uge* in EPS production. Additionally, *mpi* in modified MRS medium demonstrated negative regulation, and no significant differences between MLP and BSP were observed (Fig. 6g). Thus, we eliminated the possibility that mannose in EPS was derived from mannose-6-phosphate.

We next investigated cell aggregation and hydrophobicity (Table 2). ZW3 demonstrated cell aggregation and hydrophobicity but at lower levels than those observed in *Lb*. *kefiranofaciens* XL10. In most bacteria, the S-layer is a mesoporous surface structure composed of monomolecular protein subunits such as fibrin, fibronectin, or other collagen that adhere to the extracellular matrix^57,58^. We found four S-layer proteins in the ZW3 genome, namely, *peg*.*509*, *peg*.*1216*, *peg*.*1307*, and *peg*.*2072*. S-layer proteins have been identified in various LABs such as *Lb*. *acidophilus*, *Lb*. *helveticus*, *Lb*. *buchneri*, and *Lb*. *brevis*, with usually one or more coding genes. The larger number of S-layer proteins observed in ZW3 suggests that this strain may have more opportunity for adhesion in the GIT. Mucin-binding proteins influence colonization, and strains containing this gene specifically bind to intestinal epithelial cells and are successfully retained in the GIT. Two mucin-binding protein genes (peg.190 and peg.836) were also found in the ZW3 genome. Thus, ZW3 may specifically anchor to loci on the surface of intestinal epithelial cells.

**Table 2:** Cell surface hydrophobicity and aggregation of the investigated strains.

Name	Hydrophobicity (%)	Aggregation (%)
*Lb*. *kefiranofaciens* ZW3	14.32 ± 0.7	4.92 ± 0.2
*Lb*. *kefiranofaciens* XL10	79.9 ± 1.3	27.8 ± 2.3
*Lb*. *bulgaricus* CGMCC1.2161	18.4 ± 1.5	1.14 ± 0.3

^a^All values are shown as the mean ± S.D. of three parallel tests.

In summary, we re-annotated the sequence of *Lb*. *kefiranofaciens* ZW3 to obtain more comprehensive genome-wide information. Comparative genomic analysis of this genome was performed using other available *Lactobacillus* spp. genomes, and AAI analysis, ANI analysis, and phylogenetic construction indicated that strain DSM5016 was most closely related to ZW3. Moreover, we detected the pangenome and core genome of the strains, as well as unique genes in ZW3. Whole sequence alignment and GI analysis indicated that ZW3 evolution is driven by gene loss and gene gain as well as selective pressure on the genome. Strong self-defense mechanisms associated with survival in the GIT and dairy environments were observed in the ZW3 genome and those of other investigated strains. In terms of EPS production, four genes (*pgm*, *ugp*, *uge*, and *pgi*) were determined to encode the enzymes that regulate carbon flux through the EPS synthetic pathway, which in turn affects EPS yield. In this study, the basic genetic information and genetic relationships of ZW3 with other LAB strains were investigated, laying a theoretical foundation for future studies examining the functional properties of other LAB strains. *In vitro* and *in vivo* experiments may confirm additional functional properties of the ZW3 strain for use in the food or pharmaceutical industries.

## Methods

### Bacterial strains and growth conditions

The strains used in this study are listed in Table S2. *Lb*. *kefiranofaciens* ZW3 was grown anaerobically at 30 °C in modified MRS broth or whey medium for 24 h. Other strains were incubated in LB medium at 37 °C with shaking at 200 rpm for 16 h.

### Re-annotation of the genome of *Lb*. *kefiranofaciens* ZW3

The genome was annotated using the RAST^59^ (http://rast.nmpdr.org/) and Basys^60^ (https://www.basys.ca/) pipelines. Predictions produced by the two pipelines were compiled into a single annotation file after manual curation in the Mauve^61^ software environment. Final corrections and quality assessment of the annotation were performed with the GenePRIMP pipeline^62^. GenePRIMP was also used to identify putative pseudogenes. A circular map of the *Lb*. *kefiranofaciens* ZW3 genome was generated using Artemis and DNAPlotter software^63^.

### Comparative genomics of *Lb*. *kefiranofaciens* ZW3

The complete genome sequence of *Lb*. *kefiranofaciens* ZW3 was compared with those of *Lb*. *kefiranofaciens* DSM5016, *Lb*. *helveticus* H9, *Lb*. *helveticus* H10, *Lb*. *helveticus* R0052, and *Lb*. *acidophilus* La14 using a variety of tools. Genome sequences of the six strains were retrieved from GenBank (Table S3). To visualize conserved genomic regions or chromosomal rearrangements, whole-genome sequence alignments were performed via progressive MAUVE^64^. Estimation of the differential gene content of the genomes, as well as the whole-genome phylogeny of *Lactobacillus*, was conducted within the EDGAR software framework^65^. Venn diagrams, AAI and ANI were also designed using the EDGAR software framework.

### Other bioinformatics tools

Sequence similarity searches were performed with the BLAST suite. Whenever necessary, protein sequences were analyzed in the CDD^66^. CRISPRs were analyzed using the tools available in the CRISPRcompare web-service^67^. Similarity analysis of CRISPR spacers was undertaken using BLASTN. GIs were identified and visualized using the IslandViewer application, which utilizes three different prediction tools (IslandPick, SIGI-HMM, and IslandPath-DIMOB) relying on either sequence composition or comparative genomics^68^. Genomic regions of RM systems were determined in the REBASE genomes database^69^. The metabolic map of *Lb*. *kefiranofaciens* ZW3 was analyzed using the KEGG database. Other physical properties and structural analysis were conducted using the following online analysis software: physical properties analysis (http://web.expasy.org/protparam/), TMHMM 2.0 server for the analysis of transmembrane structure (http://www.cbs.dtu.Dk/services/TMHMM/), and secondary structure and tertiary structure analysis using PHYRE2^70^.

### Antibacterial peptide test

ZW3 was activated via inoculation into 100 mL of modified MRS medium at 4% (v/v). Three parallel lines were set. After 3 h, 3 mL of fresh culture was collected every 3 h through 36 h, then centrifuged at 8,000 *g* for 10 min. The pH values of the supernatants were adjusted to neutral, and excess catalase was added to the reaction for 2 h per mL at 37 °C to remove hydrogen peroxide. *E*. *coli* 442, *E*. *aerogenes* 435, *M*. *luteus* 444, *B*. *subtilis* 438, *S*. *enteritidis* 440, and *S*. *mutans* 377 were cultured overnight in LB medium, and a layer of solid LB medium was poured into the plates. After cooling and solidifying, 150-µL culture aliquots of indicator bacteria were spread on the plates. After the liquid had completely absorbed, sterile Oxford cups were placed on the plates, and a second layer of solid LB medium was poured, cooled, and solidified. After removal of the Oxford cups, 80 µL of treated supernatant and untreated supernatant were injected and incubated overnight at 37 °C to observe the formation of inhibition zones. Hydrogen peroxide was used as positive control.

### Acid and bile salt tolerances

Acid and bile salt tolerance were tested as previously described^14^, with modifications.

### Cell surface hydrophobicity and cell aggregation

Cell surface hydrophobicity was determined using the method of Rosenberg *et al*.^71^. Cell aggregation was tested as follows: fresh bacterial cultures in MRS broth at 30 °C were harvested, and cell pellets were washed twice with PBS and resuspended to similar adjusted cell densities (2 × 10^8^ CFU/mL). The suspensions were centrifuged (10,000 *g*, 5 min), and the pellets were resuspended in a volume of broth equal to the amount removed in the previous step. The mixtures were allowed to stand at 30 °C for 2 h. Subsequently, 1 mL of the upper suspension was assayed to measure the absorbance at wavelength of 600 nm, using broth as reference. Cellular auto-aggregation (%) was calculated as the percentage difference between the initial and final absorbance.

### Growth and EPS production curves

Seed broth was inoculated into 400 mL of basal medium at 4% (v/v). Three parallel cultures were grown at 30 °C anaerobically. Fresh samples were taken from the cultures every 8 h, and OD_600_ values were determined. The average value and standard deviation were calculated in Excel. Then, the supernatants were centrifuged at 6,000 *g* for 10 min, and the total sugar contents in the supernatants were determined using the phenol-sulfuric acid method. Reducing sugar contents in the supernatants were determined by performing 3,5-dinitrosalicylic acid (DNS) assays. EPS content in the fermentation broth was calculated as total sugar content minus reducing sugar content at each time point. Average values and standard deviations were calculated in Excel. With the fermentation time (0–88 h) as the abscissa and EPS yield and OD600 as the double vertical coordinates, growth and EPS production curves of ZW3 in modified MRS liquid medium or whey liquid medium were generated.

### Primer design and RT-qPCR of EPS production-related genes in metabolic pathways

ZW3 was cultured in modified MRS liquid medium or whey liquid medium. Cells were collected at different time points (initial logarithmic phase, mid-logarithmic phase, and stationary phase) for RNA extraction. Total RNA was prepared from cultured cells. The quantity and integrity of the purified RNA were checked by measuring the UV absorbance at wavelengths of 260 nm and 280 nm using a BioDrop-Touch device (BioDrop, Ltd., England) and by performing electrophoretic analysis, respectively. RT-PCR was conducted to determine gene expression. The RT reaction mixture (20 μL) contained 7 μL of RNA (50 pg–5 μg), 4 μL of dNTP mix (2.5 mM each), 2 μL of primer mix, 4 μL of 5× RT buffer, 2 μL of dithiothreitol (DTT) (0.1 M), and 1 μL of HiFiScript (200 U/μL) and was incubated at 42 °C for 50 min. RT products were used for PCR amplification or kept at −20 °C for RT-qPCR.

RT-PCR primers based on the five EPS production-related gene sequences of ZW3 and the 16 S rRNA sequence were designed with OligoPerfect™ Designer (http://tools.invitrogen.com/content.cfm?pageid=9716); the primers used in this study are listed in supplementary Table S3. For each primer pair, the PCR efficiency was evaluated by performing serial dilutions using a cDNA sample. The formation of primer dimers and the amplification specificity were assessed by performing melting curve analyses. The expression of various genes was analyzed using RT-qPCR with a 96-well thermocycler (C1000 Touch^TM^ Thermal Cycler, CFX96TM Real-Time System, Bio-Rad, CA, USA) with Bio-Rad CFX Manager 3.0 system software and the following conditions: 95 °C for 3 min, followed by 39 cycles of 95 °C for 10 s, a different annealing temperature for 30 s, and 72 °C for 30 s. PCR cycling was followed by melting curve analysis from 65 °C–95 °C (temperature transition rate of 0.5 °C/s) with stepwise fluorescence acquisition. The standard curve method was used in the calculations. Subsequently, the target amount was divided by the amount of 16 S rRNA to obtain a normalized target value. One of the normalized target values in the whey medium conditions was set as one. Then, each of the target values was expressed as the n-fold difference relative to the experimental control.

## Electronic supplementary material

supplementary information
