# Supplementary material for: Comparative genomics of Lactobacillus kefiranofaciens ZW3 and related members of Lactobacillus. spp reveal adaptations to dairy and gut environments
Source: Sci Rep. 2017 Oct 9;7:12827. doi: 10.1038/s41598-017-12916-0 (PMC5634458; doi:10.1038/s41598-017-12916-0)
Supplement: Supplementary file 1 — supplementary information [file 41598_2017_12916_MOESM1_ESM.pdf]

Title: Comparative genomics of *Lactobacillus kefiranofaciens* ZW3 and related members of *Lactobacillus.* spp reveal adaptations to dairy and gut environments

Author: Zhuqing Xing[1,2], Weitao Geng[1], Chao Li[1], Ye Sun[1], Yanping Wang[1]*

Name of the institute: [1]Key Laboratory of Food Nutrition and Safety, Ministry of Education, Food Engineering and Biotechnology Institute, Tianjin University of Science & Technology, Tianjin 300457, China

[2]Chinese medical college of TJUTCM, Tianjin University of Traditional Chinese Medicine, Tianjin 300193, China

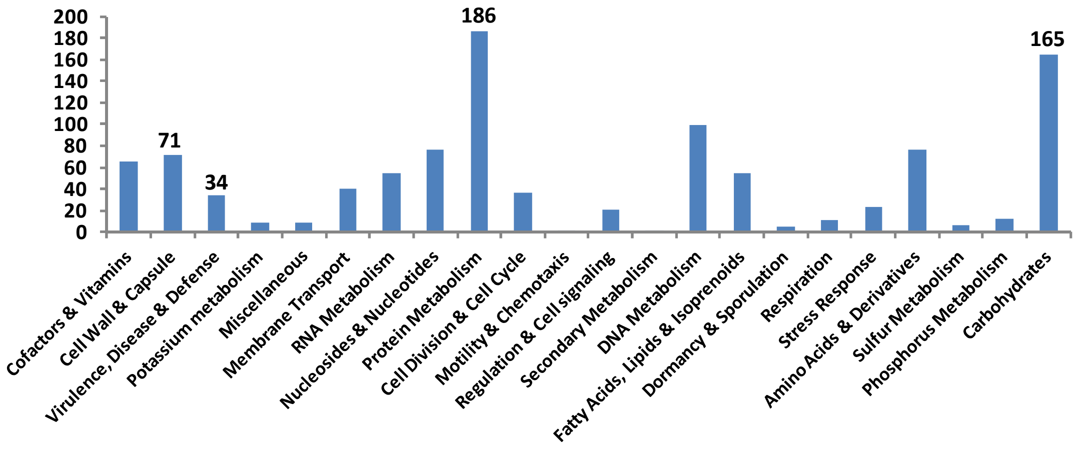

Fig. S1 Categorical distribution of the *Lactobacillus kefiranofaciens* ZW3 genome.

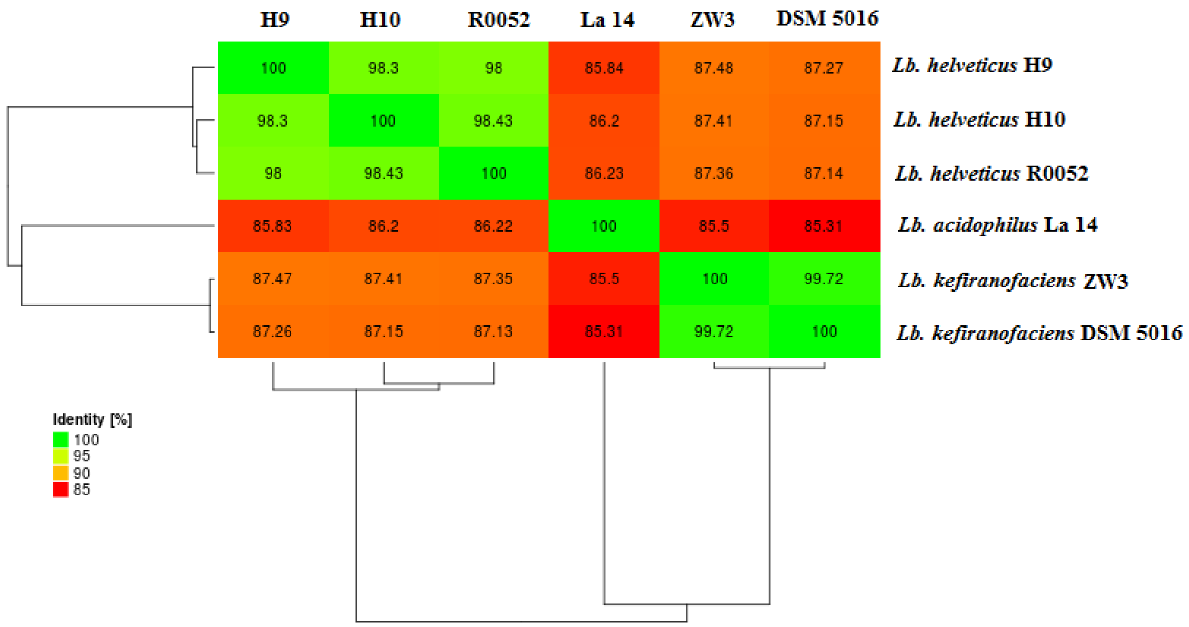

Fig. S3 Genomic heatmap based on AAI. The green colour represents a higher degree of similarity, and the red colour represents lower similarity.

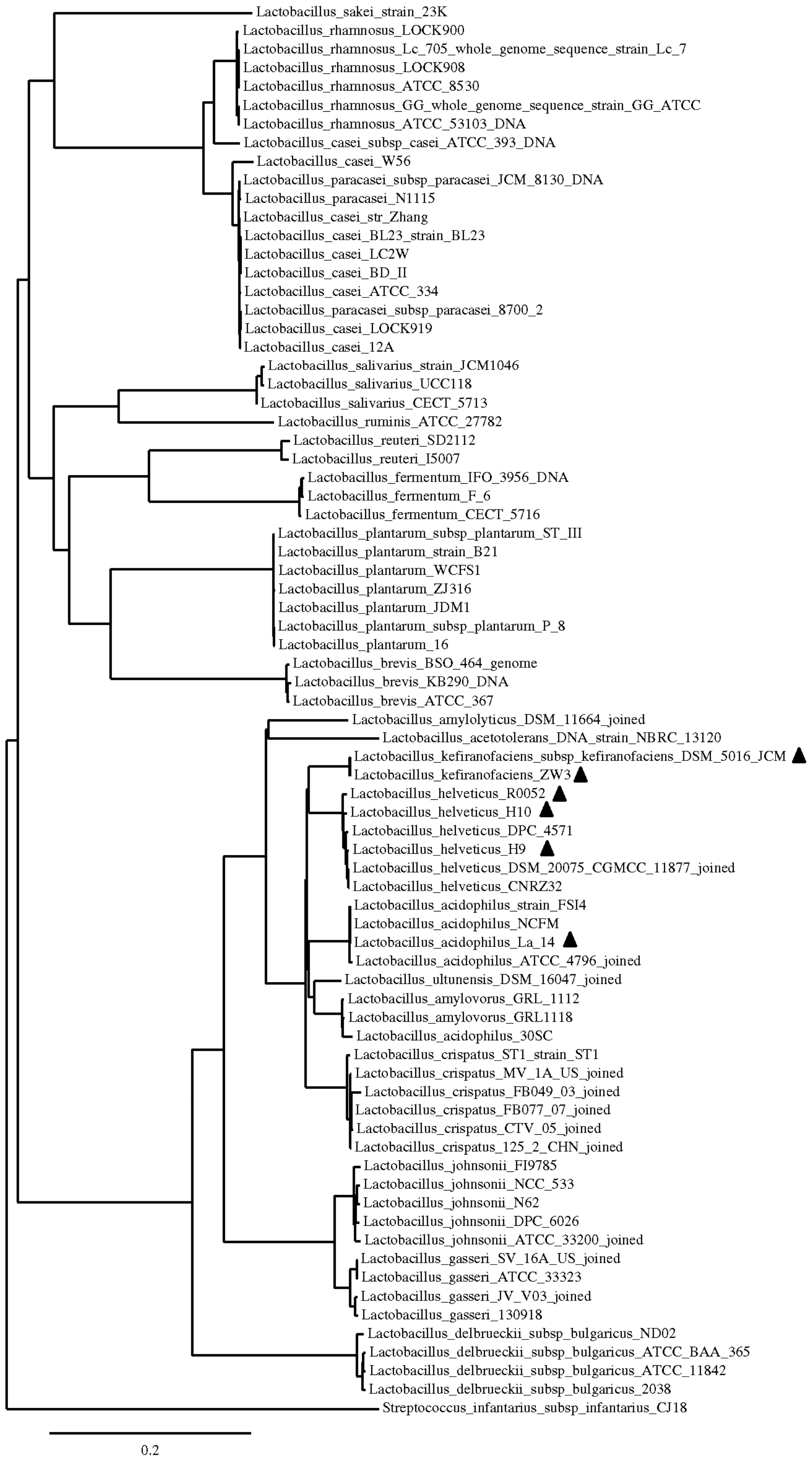

Fig. S2 Construction of a phylogenetic tree.

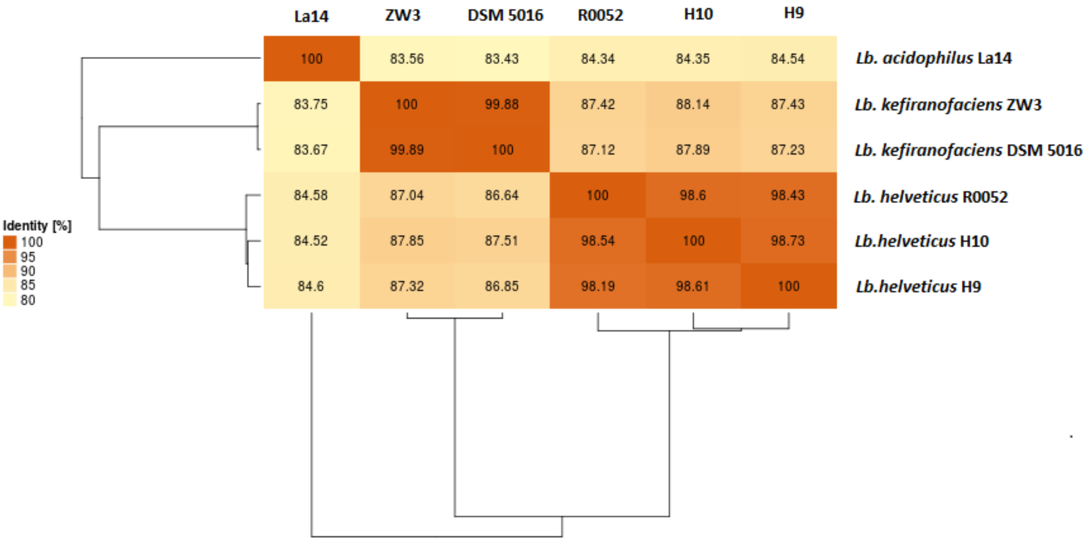

Fig. S4 Genomic heatmap based on ANI. The dark brown color represents a higher degree of similarity, and the light brown color represents lower similarity.

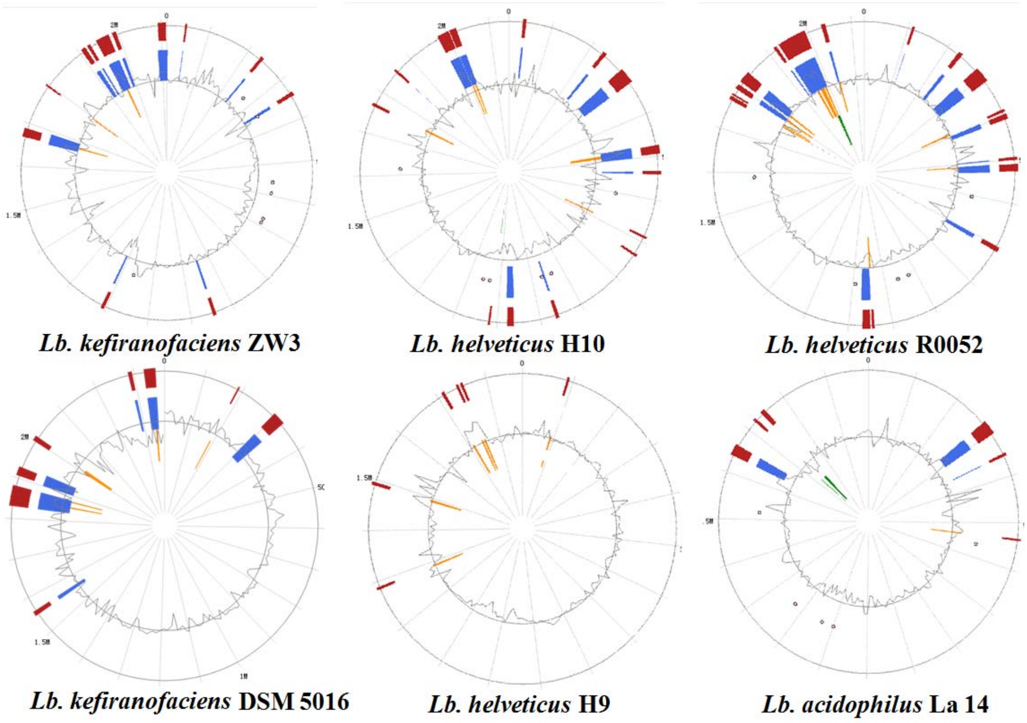

Fig. S5 GI analysis results.

(1. Predicted by at least one method (red); 2. predicted by IslandPath-DIMOB (blue); predicted by SIGI-HMM (yellow); predicted by IslandPick (green)).

Table S1 Comparison of old and new information on genes involved in sialic acid and extracellular polysaccharide synthesis

Function	Re-annotation	Previous ID	Previous annotation
EPS production	glycerol-3-phosphate cytidylyltransferase	WANG_1283	glycerol-3-phosphate cytidylyltransferase
	epsIM putative glycosyltransferase	WANG_1284	*EpsIM*, possible glycosyltransferase
	lipopolysaccharide cholinephosphotransferase *LicD1*	WANG_1285	LPS biosynthesis protein
	membrane protein involved in the export of O-antigen, teichoic acid lipoteichoic acids	WANG_1286	*EpsN*
	beta-1,3-glucosyltransferase	WANG_1287	possible glycosyltransferase *WchA*
	hypothetical protein	WANG_1288	hypothetical protein
	hypothetical protein	WANG_1289	hypothetical protein
	maltose O-acetyltransferase	WANG_1290	hypothetical protein
	2,3,4,5-tetrahydropyridine-2,6-dicarboxylate N-acetyltransferase	WANG_1291	hypothetical protein
	glycosyltransferase	WANG_1292	possible glycosyltransferase protein
	glycosyl transferase *CpsG*	WANG_1293	Glycosyl transferase family protein
	UDP-N-acetylglucosamine:LPS N-acetylglucosamine transferase	WANG_1294	UDP-N-acetylglucosamine:LPS N-acetylglucosamine transferase
	undecaprenyl-phosphate galactosephosphotransferase	WANG_1295	possible sugar-transferase
	manganese-dependent protein-tyrosine phosphatase	WANG_1296	exopolysaccharide biosynthesis protein
	tyrosine-protein kinase *EpsD*	WANG_1297	capsular polysaccharide biosynthesis protein
	tyrosine-protein kinase transmembrane modulator *EpsC*	WANG_1298	exopolysaccharide biosynthesis protein

Table S1 Comparison of old and new information on genes involved in sialic acid and extracellular polysaccharide synthesis

Function	Re-annotation	Previous ID	Previous annotation
EPS production	transcriptional regulator	WANG_1299	exopolysaccharide biosynthesis protein
sialic acid metabolism	PTS system, N-acetylglucosamine-specific IIA component	-	-
	PTS system, mannose-specific IIB component	WANG_1202	protein-N(Pi)-phosphohistidi-ne-sugar phosphotransferase
	glucosamine-1-phosphate N-acetyltransferase	WANG_1843	bifunctional protein *glmU*
	glucosamine-6-phosphate deaminase	WANG_1498	glucosamine-6-phosphate deaminase
	glucosamine--fructose-6-phosph ate aminotransferase	WANG_1196	Glutamine-fructose-6-phospha te transaminase
	UDP-N-acetylglucosamine 2-epimerase	WANG_1027	UDP-N-acetylglucosamine 2-epimerase
	PTS system, N-acetylglucosamine-specific IIB component	WANG_1036	PTS enzyme II, ABC component
	phosphoglucosamine mutase	WANG_0939	phosphoglucosamine mutase
	PTS system, N-acetylglucosamine-specific IIC component	-	-
	N-acetylglucosamine-1-phospha te uridyltransferase	WANG_1843	bifunctional protein *glmU*
	N-acetylglucosamine-6-phospha te deacetylase	WANG_1700	N-acetylglucosamine-6-P deacetylase
	sialic acid utilization regulator, *RpiR* family	WANG_1549	transcriptional regulator

Table S2 Information for experimental bacterial strains

Strains	Application	Source
L. kefiranofaciens ZW3	Subject	Laboratory stock
L. kefiranofaciens XL10	Control	Laboratory stock
L. bulgaricus CGMCC1.2161	Control	Laboratory stock
E. coli 442	Indicator strain	Laboratory stock
E. aerogenes 435	Indicator strain	Laboratory stock
M. luteus 444	Indicator strain	Laboratory stock
B. subtilis 438	Indicator strain	Laboratory stock
S. enteritidis 440	Indicator strain	Laboratory stock
S. mutans 377	Indicator strain	Laboratory stock

Table S3 Information for bacterial strains

Strains	Source	Accession No.
Lb. kefiranofaciens ZW3	Kefir grains	CP002764.1
Lb. helveticus H9	Kurut	CP002427.1
Lb. helveticus H10	Tibet traditional fermented milk	CP002429.1
Lb. helveticus R0052	Sweet acidophilus milk	CP003799
Lb. kefiranofaciens DSM5016	Kefir grains	AZGG00000000
Lb. acidophilus La14	Human	CP005926.2

Table. S3 Gene names, primers and amplification characteristics of RT-qPCR

Gene	Function	Primer sequences forward-reverse (5'-3')	Tm (°C)	Product length (bp)	Position in genome
pgi	glucose-6-phosphate isomerase	F：CAATTGCTTGCACCATTCAC<br>R：CCCTTACCAATACGCTGC	55	115	CP002764.1<br>992875-994212
pgm	a-phosphoglucose mutase	F：TCAAGTGTTCATTGCGAAGC<br>R：TTAAACCATTTGCCCGTG	55	225	CP002764.1<br>1052405-1054129
ugp	UDP-glucose pyrophosphorylase	F：ACGTCCAGGCTTTTGATTTG<br>R：GTAATGCAAGTGCCTCACG	55	201	CP002764.1<br>1120436-1121335
uge	UDP-galactose 4-epimerase	F：AAATGTGGGCGTCGATTAAG<br>R: GAAGAGTGCCCTTTCTGGTG	55	119	CP002764.1<br>302118-303110
mpi	Mannose-6-phosphate isomerase	F：TAGTCAAGGCGTGAATGGTG<br>R：TATGCAAGCAGATCCAGGTG	55	170	CP002764.1<br>999858-1000823
16S rRNA	16S ribosomal RNA	F：AAGCTGTCGCTAAAGGATG<br>R：GTCCCAATGTGGCCGATC	55	111	CP002764.1<br>1350795-1352205；<br>125360-126770；<br>142503– 143913；<br>1818497- 1819907

Table S4 Genes involved in the metabolism of different carbohydrates

Carbohydrates	Genes	R0052	H10	H9	La 14	DSM 5016	ZW3
		Number					
Lactose and galactose	Beta-galactosidase	2	2	2	2	1	1
	Lactose and galactose permease, GPH translocator family	6	6	5	1	2	3
	UDP-glucose 4-epimerase	1	1	1	1	1	1
	Galactokinase	1	1	1	1	1	1
	Aldose 1-epimerase	1	1	1	1	1	1
	Galactose operon repressor, GalR-LacI family of transcriptional regulators	1	1	1	1	1	1
	Galactose-1-phosphate uridylyltransferase	3	4	3	3	3	3
	Alpha-galactosidase	-	1	2	1	3	4
Trehalose	1,4-alpha-glucan (glycogen) branching enzyme, GH-13-type	1	-	-	1	1	1
	Trehalose phosphorylase	1	1	-	1	1	1
	PTS system, trehalose-specific IIC component	1	1	-	1	-	-
	Trehalose-6-phosphate hydrolase	1	2	-	1	-	-
	Beta-phosphoglucomutase	1	1	-	1	1	1
	Maltose-6'-phosphate glucosidase	1	1	-	1	1	1
	Trehalose operon transcriptional repressor	1	1	-	1	-	-
	PTS system, trehalose-specific IIB component	1	1	-	1	-	-
Glucoside	PTS system, cellobiose-specific IIB component	1	-	-			
	Outer surface protein of unknown function, cellobiose operon	1	-	-	3	-	-
	6-phospho-beta-glucosidase	5	-	-	8	-	-
	PTS system, cellobiose-specific IIC component	2	-	-	9	-	-

Table S4 Genes involved in the metabolism of different carbohydrates

Carbohydrates	Genes	R0052	H10	H9	La 14	DSM 5016	ZW3
		Number					
Glucose	PTS system, beta-glucoside-specific IIB component	1	-	-	5	-	-
	Beta-glucosidase	1	-	-	2	-	-
	PTS system, beta-glucoside-specific IIA component	-	-	-	4	-	-
	PTS system, cellobiose-specific IIA component	-	-	-	1	-	-
	Cellobiose phosphotransferase system YdjC-like protein	-	-	-	1	-	-
	Beta-glucoside bgl operon antiterminator, BglG family	-	-	-	3	-	-
Sucrose	Sucrose phosphorylase	2	-	-	2	1	2
	Sucrose operon repressor ScrR, LacI family	1	1	2	1	1	1
	PTS system, sucrose-specific IIC component	2	3	2	2	2	3
	PTS system, sucrose-specific IIA component	1	1	1	1	1	1
	PTS system, sucrose-specific IIB component	2	3	2	2	2	3
	Fructokinase	1	1	1	1	1	1
	Sucrose-6-phosphate hydrolase	-	-	-	1	-	-
Oligosaccharides and raffinose	Raffinose operon transcriptional regulatory protein RafR	-	-	-	1	1	1
	Alpha-galactosidase	-	-	-	1	3	4
	Sucrose phosphorylase	-	-	-	2	1	2
	Multiple sugar ABC transporter, membrane-spanning permease protein MsmG	-	-	-	2	1	2
	Glucan 1,6-alpha-glucosidase	-	-	-	1	-	-
	Beta-glucosidase	-	-	-	2	1	1

Table S4 Genes involved in the metabolism of different carbohydrates

Carbohydrates	Genes	R0052	H10	H9	La 14	DSM 5016	ZW3
		Number					
Oligosaccharides and raffinose	MSM (multiple sugar metabolism) operon regulatory protein	-	-	-	2	2	2
	Sucrose-6-phosphate hydrolase	-	-	-	1	-	-
	Multiple sugar ABC transporter, ATP-binding protein	-	-	-	2	2	2
	Multiple sugar ABC transporter, membrane-spanning permease protein MsmF	-	-	-	2	1	1
	Multiple sugar ABC transporter, substrate-binding protein	-	-	-	-	1	2
Maltose and maltodextrin	Oligo-1,6-glucosidase	1	1	-	1	1	2
	Maltose/maltodextrin ABC transporter, permease protein MalF	1	1	-	-	-	1
	Maltose-6'-phosphate glucosidase	1	1	-	1	1	2
	Neopullulanase	2	1	-	2	2	3
	Maltose/maltodextrin ABC transporter, substrate binding periplasmic protein MalE	1	1	-	1	2	2
	Glycogen phosphorylase	1	-	-	1	1	1
	Maltose phosphorylase	1	1	-	1	1	1
	Maltose O-acetyltransferase	1	1	-		1	1
	Aldose 1-epimerase	1	1	-	1	1	1
	Beta-phosphoglucomutase	1	1	-	1	1	1
	Maltodextrin glucosidase	1	1	-	1	-	1
	Maltose operon transcriptional repressor MalR, LacI family	1	1	-	1	1	1
	Multiple sugar ABC transporter, ATP-binding protein	1	1	-	2	2	2

Table S4 Genes involved in the metabolism of different carbohydrates

Carbohydrates	Genes	R0052	H10	H9	La 14	DSM 5016	ZW3
		Number					
Maltose and maltodextrin	Maltose/maltodextrin ABC transporter, permease protein MalG	-	1	-	1	1	1
	Pullulanase	-	-	-	1	-	-
	Maltose/maltodextrin transport ATP-binding protein MalK		-	-	1	-	-
	Maltose/maltodextrin ABC transporter, permease protein MalF	-	-	-	1	1	-
	Mlc, transcriptional repressor of MalT (the transcriptional activator of maltose regulon) and the manXYZ operon	-	-	-	1	-	-
